# Outcomes for FOLFIRI plus bevacizumab or cetuximab in patients treated with oxaliplatin-based adjuvant therapy: A combined analysis of FIRE-3 and CALGB/SWOG 80405 (Alliance)^☆☆,☆^

**DOI:** 10.1016/j.ejca.2025.115694

**Published:** 2025-08-05

**Authors:** Francesca Battaglin, Bert H. O’Neil, Sebastian Stintzing, Fang-Shu Ou, Tyler J. Zemla, Donna Niedzwiecki, Federico Innocenti, Howard S. Hochster, Ludwig Fischer von Weikersthal, Thomas Decker, Alexander Kiani, Ursula Vehling-Kaiser, Tobias Heintges, Christian Lerchenmüller, Lena Weiss, Kathrin Heinrich, Richard M. Goldberg, Robert J. Mayer, Richard L. Schilsky, Charles D. Blanke, Alan P. Venook, Heinz-Josef Lenz, Volker Heinemann

**Affiliations:** a USC Norris Cancer Center, Los Angeles, CA, United States; b University Hospital Grosshadern, Munich, Germany; c Department of Hematology, Oncology, and Cancer Immunology (CCM); Charité – Universitaetsmedizin Berlin, Berlin, Germany; d DKTK Heidelberg, Partner Site, Berlin, Germany; e Alliance Statistics and Data Management Center, Mayo Clinic, Rochester, MN, United States; f Department of Biostatistics and Bioinformatics and Duke Cancer Institute-Biostatistics, Duke University, Durham, NC, United States; g UNC Lineberger Cancer Center, Chapel Hill, NC, United States; h Yale Cancer Center, New Haven, CT, United States; i MVZ Gesundheitszentrum St. Marien GmbH, Amberg, Germany; j Onkologie Ravensburg, Ravensburg, Germany; k Department of Medicine IV, Klinikum Bayreuth GmbH, Bayreuth, Germany; l VK&K Studienzentrum, Landshut, Germany; m Department of Medicine II, Stadtisches Klinikum, Neuss, Germany; n Oncological Practice, Münster, Germany; o Department of Medicine III and Comprehensive Cancer Center (CCC Munich LMU), University Hospital, LMU Munich, Munich, Germany; p West Virginia University Cancer Institute, Morgantown, WV, United States; q Dana Farber Cancer Center, Boston MA, United States; r University of Chicago, Chicago IL, United States; s Oregon Health Sciences, Portland, OR, United States; t UCSF Helen Diller Cancer Center, San Francisco, CA, United States

**Keywords:** Colorectal cancer, Adjuvant chemotherapy, First-line therapy, Oxaliplatin, Bevacizumab, Cetuximab

## Abstract

**Background:**

Metastatic recurrence of colorectal cancer (mCRC) after adjuvant therapy may differ biologically from recurrence in untreated mCRC. We examined first-line treatment outcomes of patients within the phase III CALGB/SWOG 80405 (CALGB 80405) and FIRE-3 trials according to previous exposure to oxaliplatin-based adjuvant treatment.

**Methods:**

Patients from CALGB 80405 primary analysis (N =1131) and FIRE-3 intent-to-treat population (N =592) treated with FOLFIRI who previously received either oxaliplatin-based adjuvant therapy or no adjuvant therapy were identified. Progression-free survival (PFS) and overall survival (OS) were estimated by the Kaplan–Meier method and compared using the log-rank test. Adjusted Cox regression models were used to compare outcomes according to biologic agent and prior adjuvant treatment.

**Results:**

A total of 800 patients were included in the analysis. There were no significant differences in OS and PFS based on adjuvant treatment. Among patients who received adjuvant oxaliplatin-based chemotherapy (N = 123), median PFS was 11.5 months for FOLFIRI-bevacizumab (bev) and 10.1 months for FOLFIRI-cetuximab (cet) (adjusted HR [95 %CI] = 0.81 [0.56, 1.18], *p* = 0.27). Median OS in the same group was 41.0 months for bev- vs 28.5 months for cet-treated patients (adjusted HR 95 %CI] = 0.78 [0.51, 1.20], *p* = 0.26). No significant interaction between treatment arm and prior adjuvant treatment was identified.

**Conclusions:**

No statistically significant difference in either OS or PFS by biologic agent was found in this un planned subset analysis of two large, randomized phase III trials. However, further investigation is warranted to evaluate possible survival differences in larger patient cohorts

## 1. Introduction

Advances in the treatment of metastatic colorectal cancer (mCRC) have led to an increase in median patient survival which now exceeds 30 months in median in clinical trials. With median progression-free-survival (PFS) times of about 10 months for first-line treatment, subsequent therapies clearly contribute to overall survival (OS). Therefore, identifying the optimal treatment sequence is paramount in the continuum of care to achieve longer OS. The impact of prior adjuvant chemotherapy on the efficacy of the first-line treatment in the metastatic setting has not yet been investigated in depth. Most clinical trials investigating mCRC treatment strategies recruit patients who may have received prior adjuvant treatment if completed more than 6–12 months before relapse or study enrollment/randomization; however, prior oxaliplatin administration may be an exclusion criterion in some first-line trials. The outcome of this patient group is of relevance as 25–30 % of patients with stage III CRC who are treated with adjuvant oxaliplatin will eventually develop recurrence [1]. Especially in the era of targeted treatment, it is of interest whether anti-epidermal growth factor receptor (EGFR) or anti-vascular endothelial growth factor (VEGF) treatment in combination with FOLFIRI should be preferentially administered in patients pretreated with oxaliplatin in the adjuvant setting.

It has been shown that chemotherapy exposure can change tumor biology [2]. However, little is known about the specific changes induced by the treatment with 5-FU and oxaliplatin in CRC. Preclinical data suggest changes in micro-RNA [3] and induction of epithelial-mesenchymal transition of the tumor cells in response to oxaliplatin-based chemotherapy treatment [4]. This could especially influence the efficacy of anti-EGFR therapy. Of note, several resistance mechanisms have been suggested to explain oxaliplatin resistance in mCRC, including upregulation of excision repair cross complementing group 1 (ERCC1), cellular transporters and thymidylate synthase (TS), deregulation of the NF-KB pathway, and epigenetic silencing of the *SRBC* gene encoding for a protein that interacts with BRCA1, but no biomarker currently exists for patient selection [5].

In addition to tumor evolution driven by adjuvant treatment, tumor heterogeneity within the cells that make up the recurrent tumor may help to explain differences in outcomes in the palliative treatment setting.

In this study, we aimed to evaluate the impact of previous adjuvant oxaliplatin treatment on the efficacy of first-line FOLFIRI plus cetuximab (cet) and FOLFIRI plus bevacizumab (bev), leveraging individual patient data from two large randomized phase III trials: Cancer and Leukemia Group B (CALGB)/SWOG 80405 (CALGB 80405) (Alliance) and FIRE-3 (AIO KRK-0306) [6–8].

## 2. Patients and methods

### 2.1. Study population

**Fig. 1.**
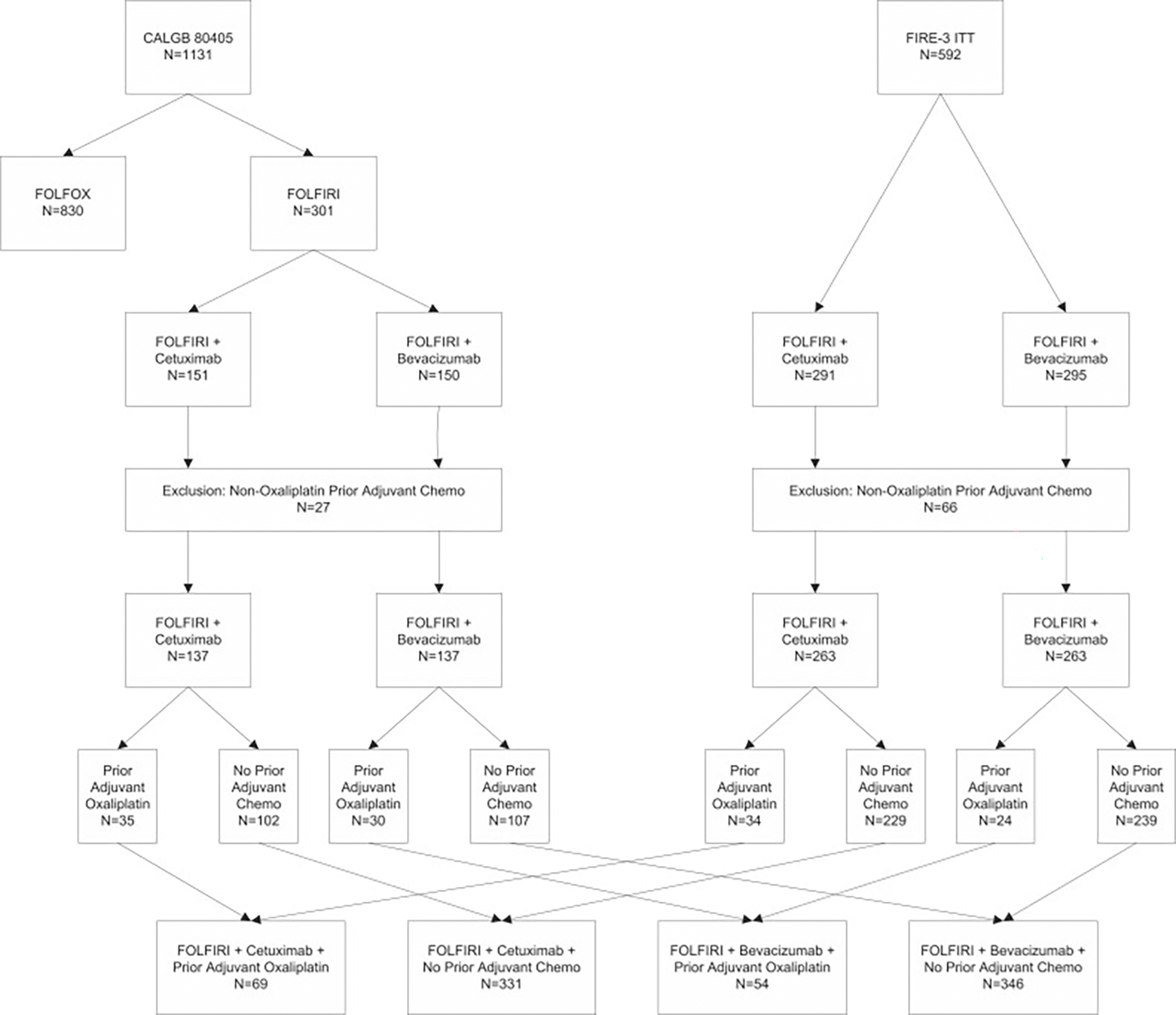
Study CONSORT diagram.

Patients from the CALGB 80405 primary analysis (N =1131) and the FIRE-3 trial intention-to-treat population (N =592) treated with FOLFIRI chemotherapy backbone and who had received either adjuvant oxaliplatin-based therapy or no adjuvant therapy were identified for this analysis. Patients who had received a prior adjuvant treatment without oxaliplatin (N =93, total from both CALGB 80405 and FIRE-3) were excluded (Study CONSORT diagram - Fig. 1). Patient demographics are reported in Table 1.

The FIRE-3 trial investigated the efficacy of FOLFIRI plus cet vs FOLFIRI plus bev as first-line treatment for mCRC [6,8]. Patients were required to be untreated for metastatic disease, but a prior adjuvant chemotherapy was allowed if it was terminated at least 6 months prior to study randomization. The study protocol was amended in 2008 to restrict entry to patients with *KRAS* exon 2 wild-type (codons 12 and 13) tumors (intention-to-treat population). Full study protocol and treatment schedule have been previously described [6]. The trial had been registered at ClinicalTrials.gov as NCT00433927.

The CALGB 80405 trial was conducted to compare the efficacy of first-line chemotherapy (FOLFOX or FOLFIRI, based on investigator choice before randomization) plus either cet, bev, or a combination of cet and bev in advanced CRC and mCRC [7]. Patients were required to be untreated for metastatic disease, but prior adjuvant chemotherapy was allowed if terminated at least 12 months prior to randomization. Three years after the start of the trial, *KRAS* wild-type (codons 12 and 13) status became an eligibility criterion, and the dual antibody arm was discontinued because of lack of efficacy. Full study protocol and treatment schedule have been previously described [7]. The trial had been registered at ClinicalTrials.gov as NCT00265850. CALGB is now part of the Alliance for Clinical Trials in Oncology.

Both studies were conducted in accordance with guidelines of the Declaration of Helsinki and Good Clinical Practice. Patients from the CALGB 80405 and FIRE-3 trials provided written informed consent prior to study enrollment. The study protocols were approved by the institutional review board of each participating institution.

### 2.2. Statistical methods

The outcomes of interest of this study were OS, defined as time from study entry until death, and PFS, defined as time from study entry until first documented progression or death. Surviving patients were censored at their last known follow-up for OS. Patients alive and without documented disease progression were censored at the most recent tumor assessment for PFS. Overall response rate (ORR), per RECIST 1.0, was also examined.

Patients treated in CALGB 80405 and FIRE-3 were combined within each subgroup for the outcome analyses. Continuous variables were presented as medians with interquartile percentiles, whereas categorical variables were expressed as percentages. We used the Kruskal-Wallis H test to test differences in continuous baseline characteristics with treamtent subgroup as independent factors (levels: No Prior Adjuvant + Bev, No Prior Adjuvant + Cet, Prior Adjuvant Oxaliplatin + Bev, Prior Adjuvant Oxaliplatin + Cet). The Pearson’s Chi-squared test was used to determine the association between categorical baseline characteristics and treatment subgroup. The distribution of OS and PFS within treatment subgroups were estimated by the Kaplan–Meier method and compared using the log-rank test using PROC LIFETEST in SASv9.4. Multivariable Cox regression models were used for OS and PFS comparing outcomes according to biologic agent and prior adjuvant treatment while adjusting for study (CALGB 80405 vs FIRE-3), age (continuous), treatment arm (bev vs cet), sex (male vs female), number of metastatic sites (1 vs 2 vs 3 +), primary tumor status (in-place vs not), and sidedness (right/transverse vs left) utilizing PROC PHREG in SAS v9.4.

All analyses were performed using SAS version 9.4 with statistical significance assessed at the 0.05 alpha level.

## 3. Results

A total of 800 patients treated with first-line FOLFIRI chemotherapy were included in the study (Figure 1). Median (95 % CI) follow-up for OS is 31.4 (26.5, 34.2) months for CALGB 80405 (data cut-off on January 18, 2018) and 26.4 (24.0, 28.7) months for FIRE-3 (data cut-off January 21, 2015). 123 patients received adjuvant oxaliplatin-based treatment (N = 65 from CALGB 80405 and N = 58 from FIRE-3), whereas 677 had received no adjuvant treatment (N = 209 from CALGB 80405 and N = 468 from FIRE-3). 400 patients across both trials were randomized to receive bev out of which 54 (13.5 %) had prior adjuvant oxaliplatin treatment, and 400 to receive cet including 69 patients (17.3 %) in the prior adjuvant oxaliplatin group.

The distributions of age, primary tumor location, and number of metastatic sites were comparable between treatment subsets, while the percentage of female patients was higher in the prior adjuvant oxaliplatin group compared to no prior adjuvant (Table 1). Prior resection of the primary tumor and prior pelvic radiation therapy were significantly more frequent in patients who received prior adjuvant treatment, as expected.

**Fig. 2.**
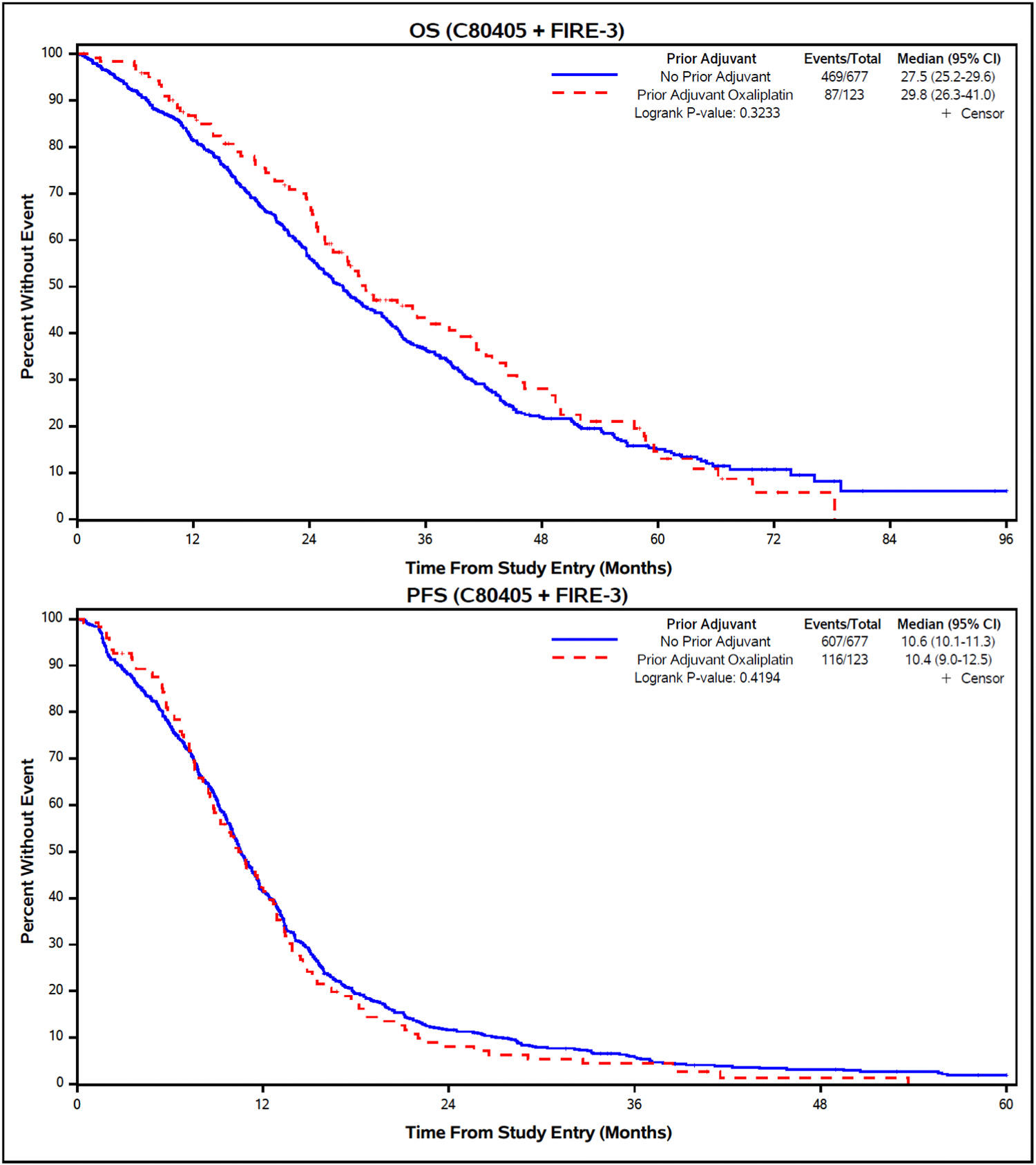
OS and PFS according to previous adjuvant oxaliplatin treatment (combined analysis). Abbreviations: OS, overall survival; PFS, progression-free-survival.

**Fig. 3.**
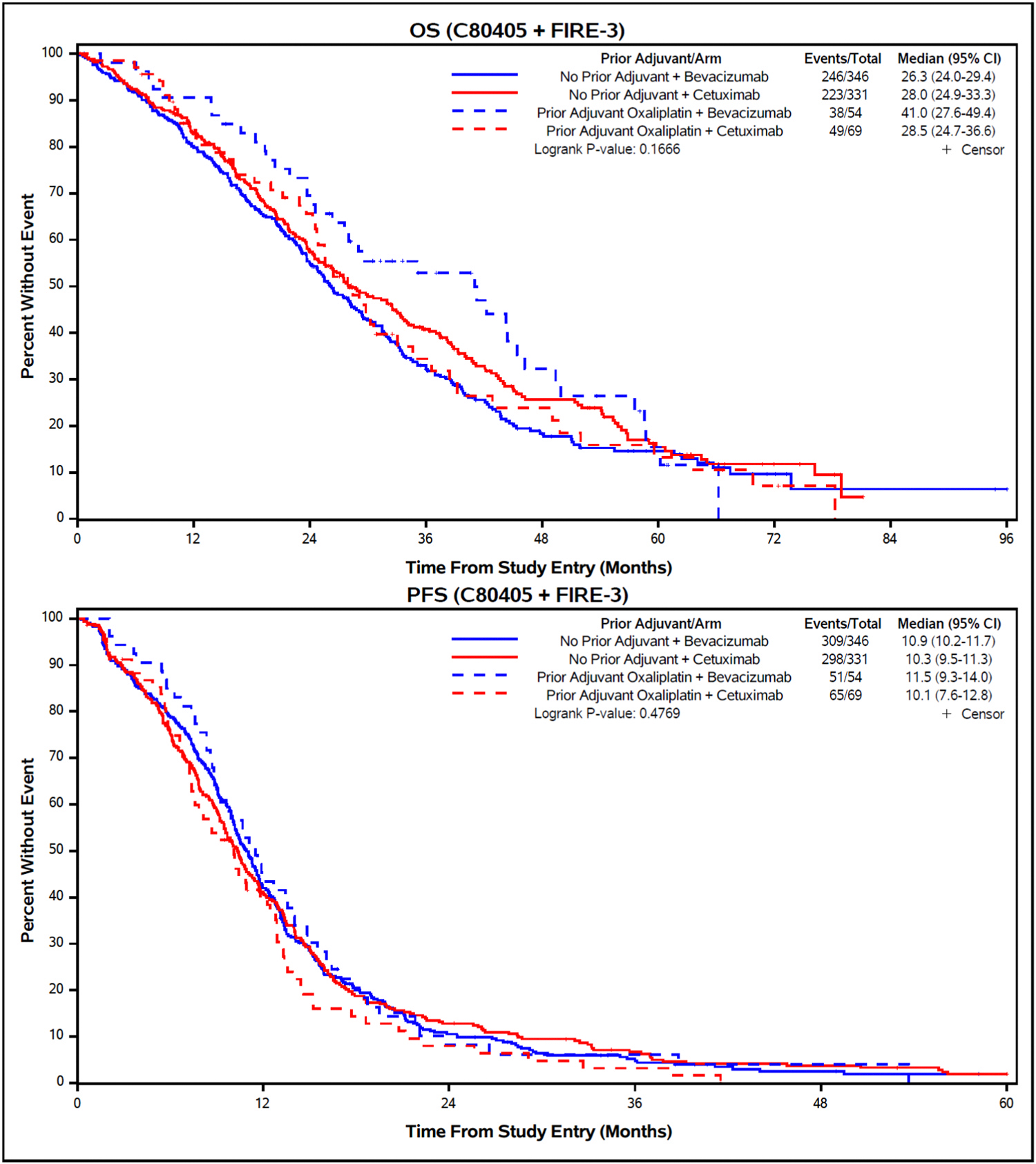
OS and PFS according to previous adjuvant oxaliplatin treatment and biologic agent (combined analysis). Abbreviations: OS, overall survival; PFS, progression-free-survival.

In the combined treatment analysis, there were no significant differences in OS (median 29.8 vs 27.5 months, unadjusted HR (95 % confidence interval [CI]) = 0.89 [0.71, 1.12]; logrank *p* = 0.32) or PFS (median 10.4 vs 10.6 months, unadjusted HR [95 % CI] = 1.09 [0.89, 1.32]; logrank *p* = 0.42) in patients who received prior adjuvant oxaliplatin vs no adjuvant treatment (Fig. 2). No statistically significant differences were observed across the combination of previous adjuvant treatment (yes/no) and biologic agents that the patients received (bev/cet) (OS logrank *p* = 0.17 and PFS logrank *p* = 0.48, respectively) (Fig. 3). Among patients who received adjuvant oxaliplatin-based chemotherapy median PFS was 11.5 months for bev and 10.1 months for cet (adjusted HR [95 % CI] = 0.81 [0.56, 1.18], *p* = 0.27). However, median OS in the same group was 41.0 months for bev vs 28.5 months for cet, although this difference did not reach statistical significance (adjusted HR [95 % CI] = 0.78 [0.51, 1.20], *p* = 0.26).

No significant differences were observed in ORR, although a numerically higher ORR was observed in patients treated with cet vs bev after receiving adjuvant oxaliplatin (58 % vs 46.3 %) (Table 2). No significant interaction between treatment arm and prior oxaliplatin adjuvant treatment was identified for either PFS or OS in multivariable analyses (Table 3).

Results from individual trials were comparable to those observed in the combined analyses (Supplementary material Figures S1–S4).

## 4. Discussion

Despite the fact that adjuvant therapy was permitted for trial inclusion, a small number of prior 5-FU or capecitabine plus oxaliplatin-treated patients were enrolled in both CALGB 80405 and FIRE-3 clinical trials. It is estimated that about 20 % of patients present with mCRC at diagnosis, and up to 44 % of those initially diagnosed with localized disease will eventually develop metastases [9]. Adjuvant oxaliplatin plus a fluoropyrimidine chemotherapy is the standard of care for patients with stage III CRC; however, about 30 % of these patients will experience disease recurrence despite adjuvant treatment [10]. Accordingly, the population of patients who start a first-line treatment having received prior adjuvant chemotherapy is likely higher than what we observed in FIRE-3 and CALGB 80405, where about 10–20 % of all patients received oxaliplatin treatment prior to entering the trial. Nevertheless, these data are in line with previous reports from other major first-line trials conducted around the same time, where only 10–15 % of patients were exposed to a prior adjuvant treatment [11–14]. It has to be noted that not all patients treated with prior adjuvant chemotherapy meet the inclusion criteria for first-line trials. Part of this is due to the eligibility criteria that may specify a permissible time interval between the last administration of adjuvant treatment and cancer recurrence or study randomization. This is meant to avoid the enrollment of patients whose tumor progressed while they were or shortly after they completed adjuvant chemotherapy on the assumption that this indicates that their tumors were chemotherapy resistant. On the other hand, eligibility specifications often allow treatment with prior single agent fluoropyrimidine, as opposed to oxaliplatin plus a fluoropyrimidine as adjuvant therapy, in first-line trials investigating oxaliplatin-containing regimens. In the CALGB 80405 study, where the chemotherapy backbone was based on investigators choice before randomization, all treating physicians made the pragmatic decision to not administering FOLFOX in those cases where oxaliplatin-based adjuvant chemotherapy had failed.

Synchronous mCRC has been shown to be associated with poorer survival compared to solitary CRC in several studies [15–17]. On the other hand, when metachronous mCRC develops in patients previously exposed to adjuvant chemotherapy, acquired treatment resistance may be a significant challenge. The introduction of oxaliplatin-based adjuvant regimens has lowered recurrence risks and increased survival, including survival after relapse (SAR), in stage III CRC [18]. However, the influence of prior adjuvant treatment on the efficacy of first-line trials is often not reported and data differentiating between fluoropyrimidine monotherapy and oxaliplatin-based adjuvant regimens are often missing. In particular, virtually no data are available for the first-line use of chemotherapy plus either an anti-EGFR compared to an anti-VEGF targeted monoclonal antibody in this subgroup of patients. In our unplanned exploratory subset analysis of FIRE-3 and CALGB 80405 no significant differences in OS, PFS, or ORR by biologic agent were found between patients treated with FOLFIRI chemotherapy backbone who either received oxaliplatin in the adjuvant setting and those who had no adjuvant treatment. However, a numerically longer OS favoring bev vs cet was observed in patients previously exposed to adjuvant oxaliplatin. The small number of patients in these treatment subgroups (N = 54 and N = 69, respectively) and limited statistical power may help to explain the lack of statistical significance. The large number attrition in our study population may also have affected this result, hence further confirmation of this finding in larger cohorts is warranted before drawing any conclusion. Nevertheless, growing evidence suggests that treatment efficacy may be affected by complex functional interactions with tumor biology and tumor microenvironment (TME) [19–21]. A possibly decreased efficacy of cet in oxaliplatin pretreated patients may be explained in part by a transition of the CRC cells from an epithelial type to a mesenchymal type which is less dependent on an activated EGFR pathway due to TGFß activation [22]. TGFß can also inhibit cet-mediated antibody-dependent cell-mediated cytotoxicity and suppress anti-tumor immune response [23,24]. As the mesenchymal type of CRC has angiogenesis as a major driver of carcinogenesis, anti-VEGF treatment may be more effective in this subgroup. This possibility has been described before in preclinical models [4] but clinical validation is missing so far.

On the other hand, conflicting evidence favors the combination of cet over bev with FOLFIRI in consensus-molecular subgroup 4 CRC (characterized by epithelial mesenchymal transition and activated TGFβ signaling) [19], hence more complex biologic mechanisms and interaction between chemotherapy treatment, biologic agents, tumor, and TME may explain the trend that we observed after adjuvant oxaliplatin treatment. Another possible explanation could be the modification of micro-RNA (miRNA) expression profiles following previous chemotherapy [3]. Data are accumulating on the role of miRNA and non-coding RNAs in tumor progression and treatment resistance, including targeted agents in mCRC [25,26]. A 10-miRNA panel signature has been recently reported as a biomarker to predict risk of recurrence and response to oxaliplatin-based adjuvant treatment in stage II-III CRC [27]. However, whether changes in miRNA expression levels related to adjuvant treatment may impact first-line treatment outcomes at disease recurrence has yet to be established. Additionally, tumor heterogeneity leading to metastases that are biologically different from the dominant clone in the primary tumor could provide another possible explanation for a difference in behavior in the metastatic setting [28, 29]. In fact, selective pressure from primary treatments may promote the emergence of resistant subclones with survival advantages and different molecular profiles that can impact subsequent treatment response [30,31].

Limitations of this study are the small number of patients who received adjuvant treatment within the CALGB 80405 and FIRE-3 trial, and the molecular selection limited to *KRAS* exon 2 wild-type (codons 12 and 13) tumors. No further molecular stratification was possible due to the small sample size in the treatment subsets of interest. Furthermore, patients treated with first-line FOLFOX chemotherapy backbone within CALGB 80405 were not included in the analysis and none of these patients received oxaliplatin-based adjuvant treatment, hence no data are available in this patient subset.

In conclusion, although no statistically significant outcome differences by biologic agent were found in this unplanned subset analysis of the CALGB 80405 and FIRE-3 trials, prior adjuvant oxaliplatin chemotherapy may have the ability to affect mCRC tumor biology and its effect on first-line clinical trial treatments in mCRC should be further investigated.

## Supplementary Material

Supplemental Material

## Figures and Tables

**Table 1:** Patient baseline characteristics.

	No Prior Adjuvant + Bev (N = 346)	No Prior Adjuvant + Cet (N = 331)	Prior Adj Oxaliplatin + Bev (N = 54)	Prior Adj Oxaliplatin + Cet (N = 69)	Total (N = 800)	*P*-value

**Study**						**< 0.0001**
CALGB 80405	107 (30.9 %)	102 (30.8 %)	30 (55.6 %)	35 (50.7 %)	274 (34.3 %)	
FIRE-3	239 (69.1 %)	229 (69.2 %)	24 (44.4 %)	34 (49.3 %)	526 (65.8 %)	
**Age (yrs)**						0.4370
N	346	331	54	69	800	
Mean (SD)	61.4 (9.9)	61.5 (9.6)	62.6 (12.2)	61.5 (7.6)	61.5 (9.8)	
Median	63.0	62.0	67.0	61.0	62.5	
Q1, Q3	55.0, 69.0	55.0, 70.0	56.0, 71.0	56.0, 67.0	55.0, 69.0	
Range	(31.0–81.0)	(36.0–81.0)	(27.0–81.0)	(43.0–78.0)	(27.0–81.0)	
**Tumor Location (sidedness)**						0.1664
Missing	17	19	2	2	40	
Left	226 (68.7 %)	237 (76.0 %)	36 (69.2 %)	45 (67.2 %)	544 (71.6 %)	
Right/Transverse	103 (31.3 %)	75 (24.0 %)	16 (30.8 %)	22 (32.8 %)	216 (28.4 %)	
**Number of Metastatic Sites**						0.1915
Missing	1	3	0	0	4	
1	136 (39.4 %)	140 (42.7 %)	31 (57.4 %)	35 (50.7 %)	342 (43.0 %)	
2	138 (40.0 %)	119 (36.3 %)	16 (29.6 %)	21 (30.4 %)	294 (36.9 %)	
3 +	71 (20.6 %)	69 (21.0 %)	7 (13.0 %)	13 (18.8 %)	160 (20.1 %)	
**Gender**						**0.0332**
Male	222 (64.2 %)	235 (71.0 %)	31 (57.4 %)	39 (56.5 %)	527 (65.9 %)	
Female	124 (35.8 %)	96 (29.0 %)	23 (42.6 %)	30 (43.5 %)	273 (34.1 %)	
**Prior Pelvic Radiation Therapy**						**< 0.0001**
Missing	1	1	0	0	2	
No	329 (95.4 %)	324 (98.2 %)	38 (70.4 %)	46 (66.7 %)	737 (92.4 %)	
Yes	16 (4.6 %)	6 (1.8 %)	16 (29.6 %)	23 (33.3 %)	61 (7.6 %)	
**Primary Tumor In-place**						**< 0.0001**
Missing	2	3	0	0	5	
No	266 (77.3 %)	246 (75.0 %)	54 (100.0 %)	68 (98.6 %)	634 (79.7 %)	
Yes	78 (22.7 %)	82 (25.0 %)	0 (0.0 %)	1 (1.4 %)	161 (20.3 %)	

**Table 2:** Response evaluation.

	No Prior Adjuvant + Bev (N = 346)	No Prior Adjuvant + Cet (N = 331)	Prior Adj Oxaliplatin + Bev (N = 54)	Prior Adj Oxaliplatin + Cet (N = 69)	Total (N = 800)	*P*-value

**Best Response**						**0.0010**
CR	9 (2.8 %)	19 (6.6 %)	3 (5.9 %)	7 (10.8 %)	38 (5.3 %)	
PR	201 (63.6 %)	182 (63.4 %)	22 (43.1 %)	33 (50.8 %)	438 (60.9 %)	
SD	82 (25.9 %)	63 (22.0 %)	25 (49.0 %)	18 (27.7 %)	188 (26.1 %)	
PD	24 (7.6 %)	23 (8.0 %)	1 (2.0 %)	7 (10.8 %)	55 (7.6 %)	
Missing	30	44	3	4	81	
**Overall Response Rate**						0.2228
No	136 (39.3 %)	130 (39.3 %)	29 (53.7 %)	29 (42.0 %)	324 (40.5 %)	
Yes	210 (60.7 %)	201 (60.7 %)	25 (46.3 %)	40 (58.0 %)	476 (59.5 %)	

Abbreviations: Bev, bevacizumab; Cet, cetuximab; CR, complete response; PD, progressive disease; PR, partial response; SD, stable disease.

**Table 3:** Adjusted Cox models for OS and PFS according to previous adjuvant oxaliplatin treatment and biologic agent.

					*P*-Values		
	
Outcome		HR	Lower CI	Upper CI	Prognostic	Comparsion	Interaction:Arm (Bev/Cet) *Prior Adjuvant (Oxaliplatin/None)

**OS**	**Prior Adjuvant Oxaliplatin vs No Prior Adjuvant**	0.91	0.72	1.1	0.46		0.14
	Prior Adjuvant Oxaliplatin vs No Prior Adjuvant in Bevacizumab	0.76	0.54	1.08		0.13	
	Prior Adjuvant Oxaliplatin vs No Prior Adjuvant in Cetuximab	1.08	0.78	1.50		0.63	
	Bevacizumab vs Cetuximab in No Prior Adjuvant	1.11	0.92	1.34		0.27	
	Bevacizumab vs Cetuximab in Prior Adjuvant Oxaliplatin	0.78	0.51	1.20		0.26	
**PFS**	**Prior Adjuvant Oxaliplatin vs No Prior Adjuvant**	1.16	0.94	1.43	0.18		0.42
	Prior Adjuvant Oxaliplatin vs No Prior Adjuvant in Bevacizumab	1.06	0.78	1.44		0.71	
	Prior Adjuvant Oxaliplatin vs No Prior Adjuvant in Cetuximab	1.25	0.94	1.66		0.12	
	Bevacizumab vs Cetuximab in No Prior Adjuvant	0.96	0.81	1.13		0.62	
	Bevacizumab vs Cetuximab in Prior Adjuvant Oxaliplatin	0.81	0.56	1.18		0.27	

Model adjusted for: Study, Age, Arm, Sex, No. of Met. Sites, Primary Tumor Status, and Sidedness Abbreviations: OS, overall survival; PFS, progression-free-survival.

## Data Availability

Deidentified patient data and data dictionary are available via NCTN data archive (https://nctn-data-archive.nci.nih.gov/) or Alliance data sharing (https://www.allianceforclinicaltrialsinoncology.org/main/public/standard.xhtml?path=%2FPublic%2FDatasharing).

## Appendix A. Supporting information

Supplementary data associated with this article can be found in the online version at doi:10.1016/j.ejca.2025.115694.
