# Supplementary material for: Outcomes for FOLFIRI plus bevacizumab or cetuximab in patients treated with oxaliplatin-based adjuvant therapy: A combined analysis of FIRE-3 and CALGB/SWOG 80405 (Alliance)
Source: Eur J Cancer. Author manuscript; Available in PMC 2026 Apr 13. (PMC13075548; doi:10.1016/j.ejca.2025.115694)

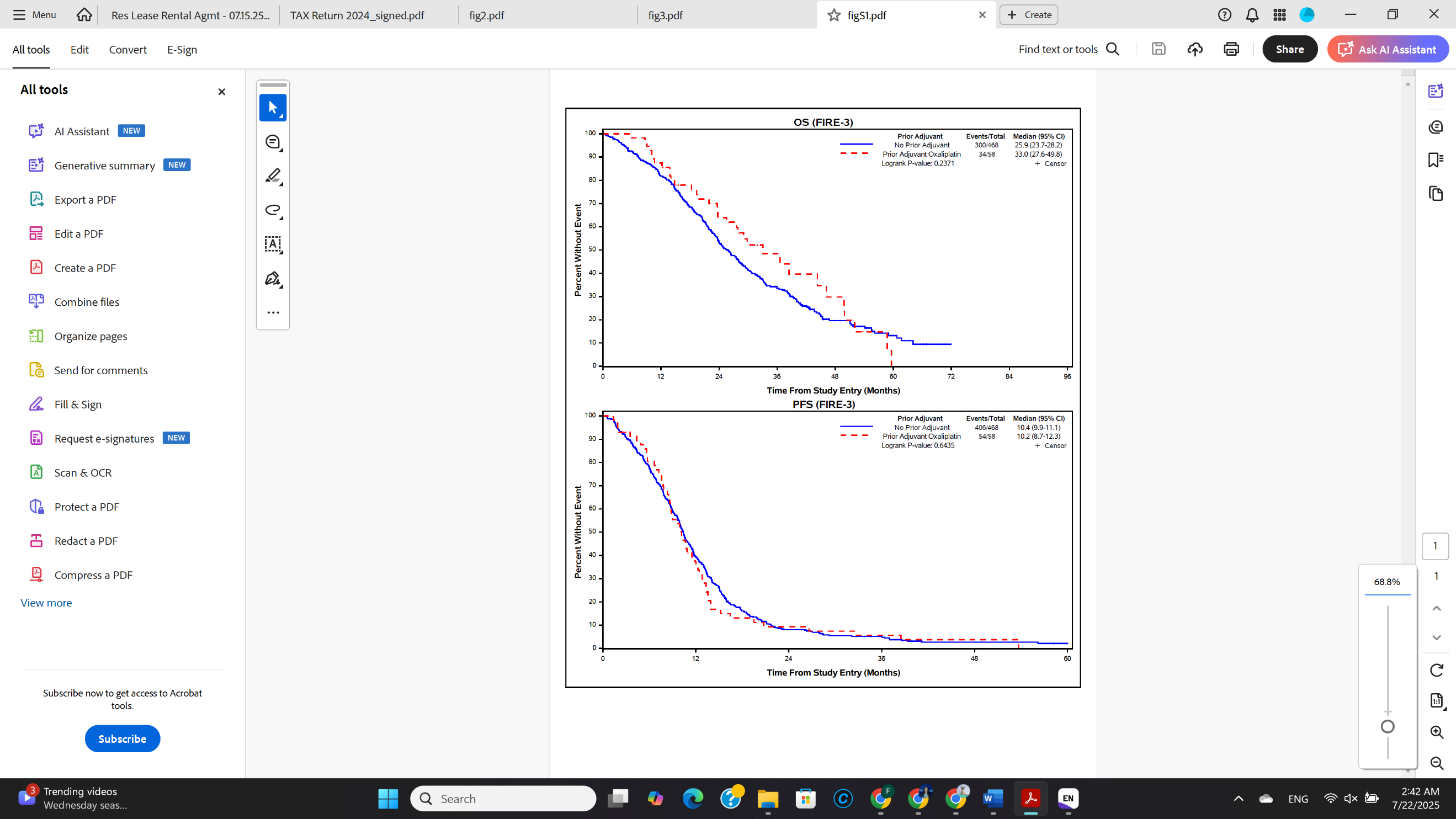
**Figure S1. OS and PFS according to previous adjuvant oxaliplatin treatment (FIRE-3).**

**Figure S2. OS and PFS according to previous adjuvant oxaliplatin treatment (CALGB 80405).**


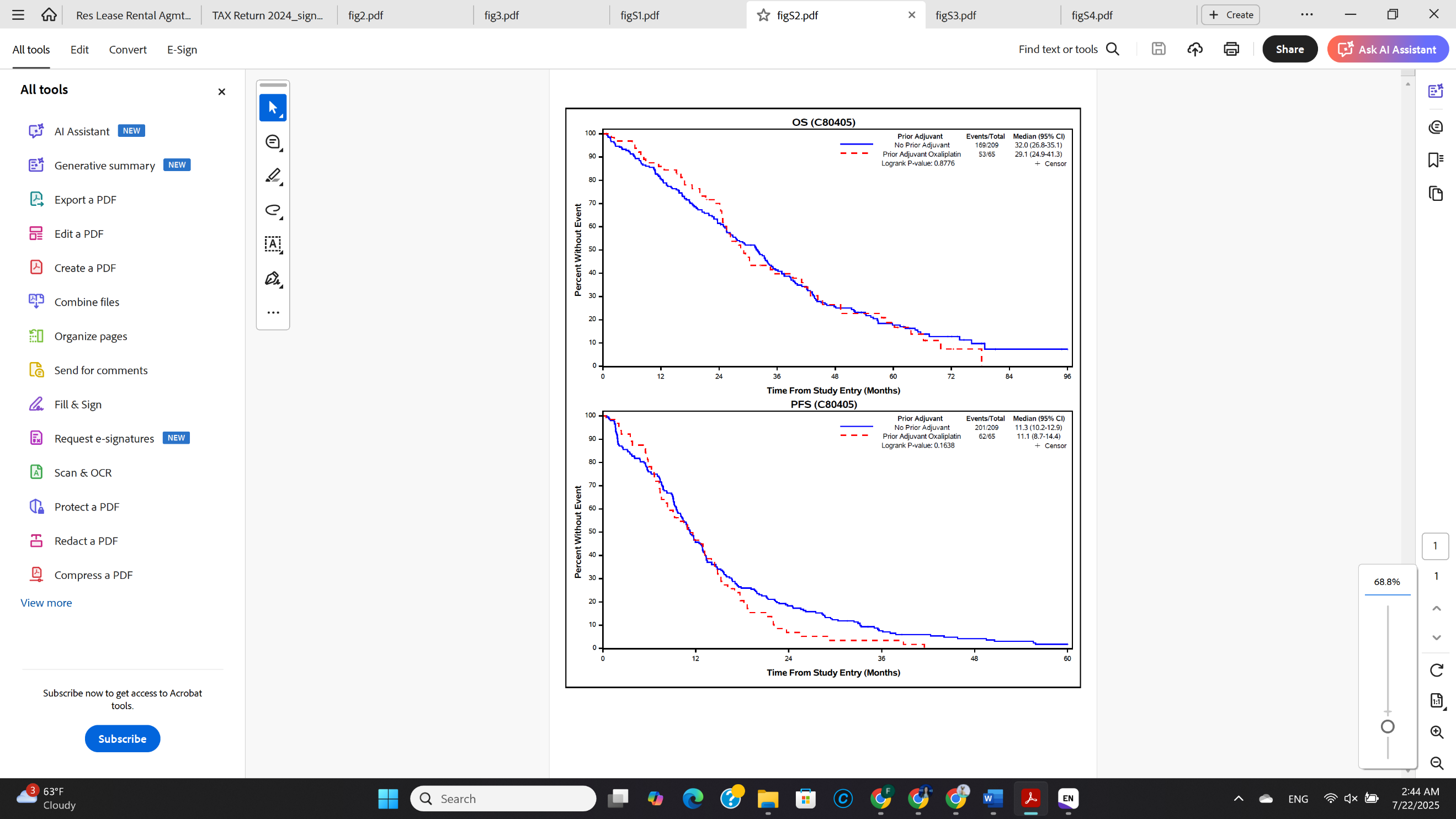


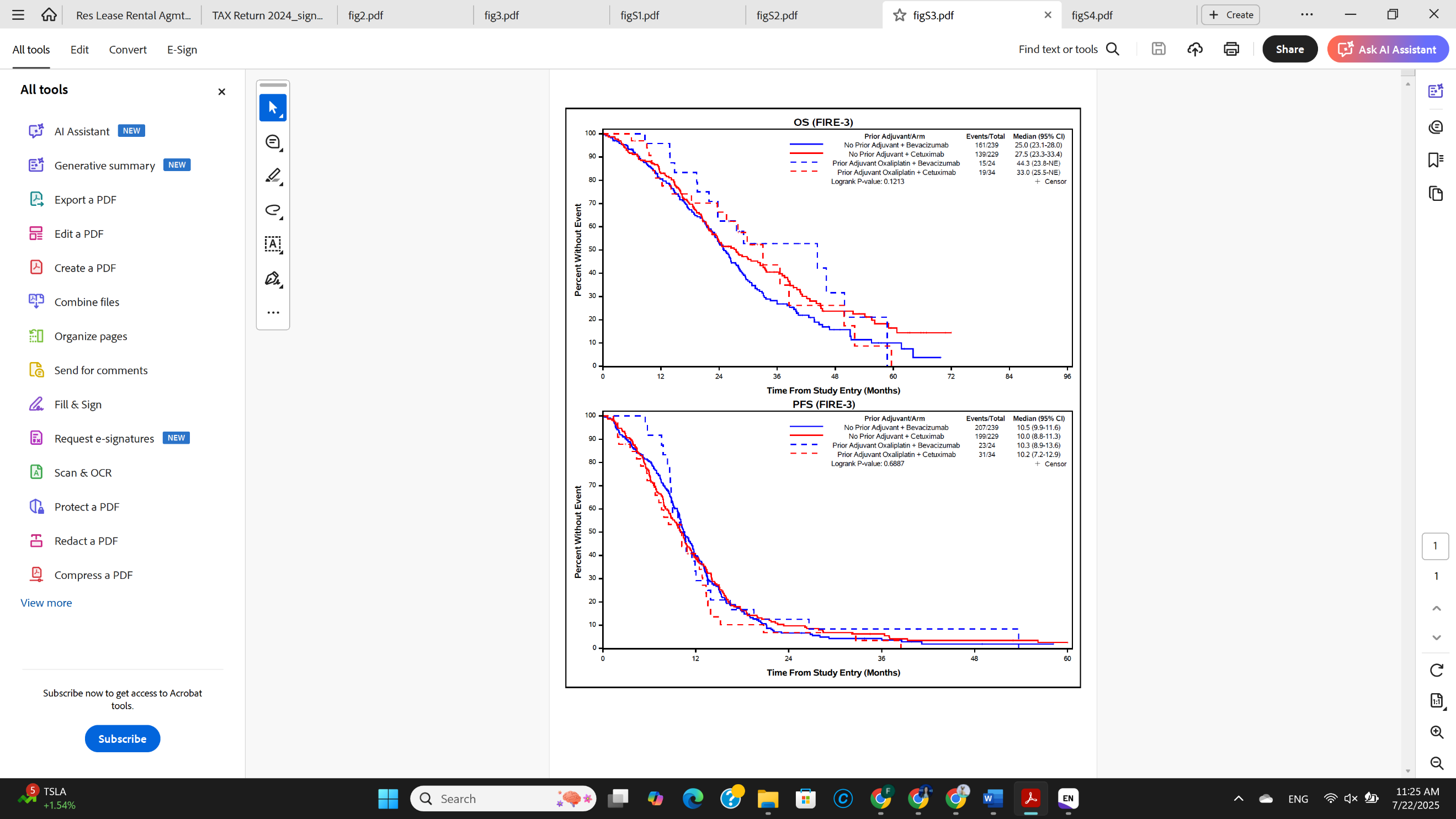
**Figure S3. OS and PFS according to previous adjuvant oxaliplatin treatment and biologic agent (FIRE-3).**

**Figure S4. OS and PFS according to previous adjuvant oxaliplatin treatment and biologic agent (CALGB 80405).**


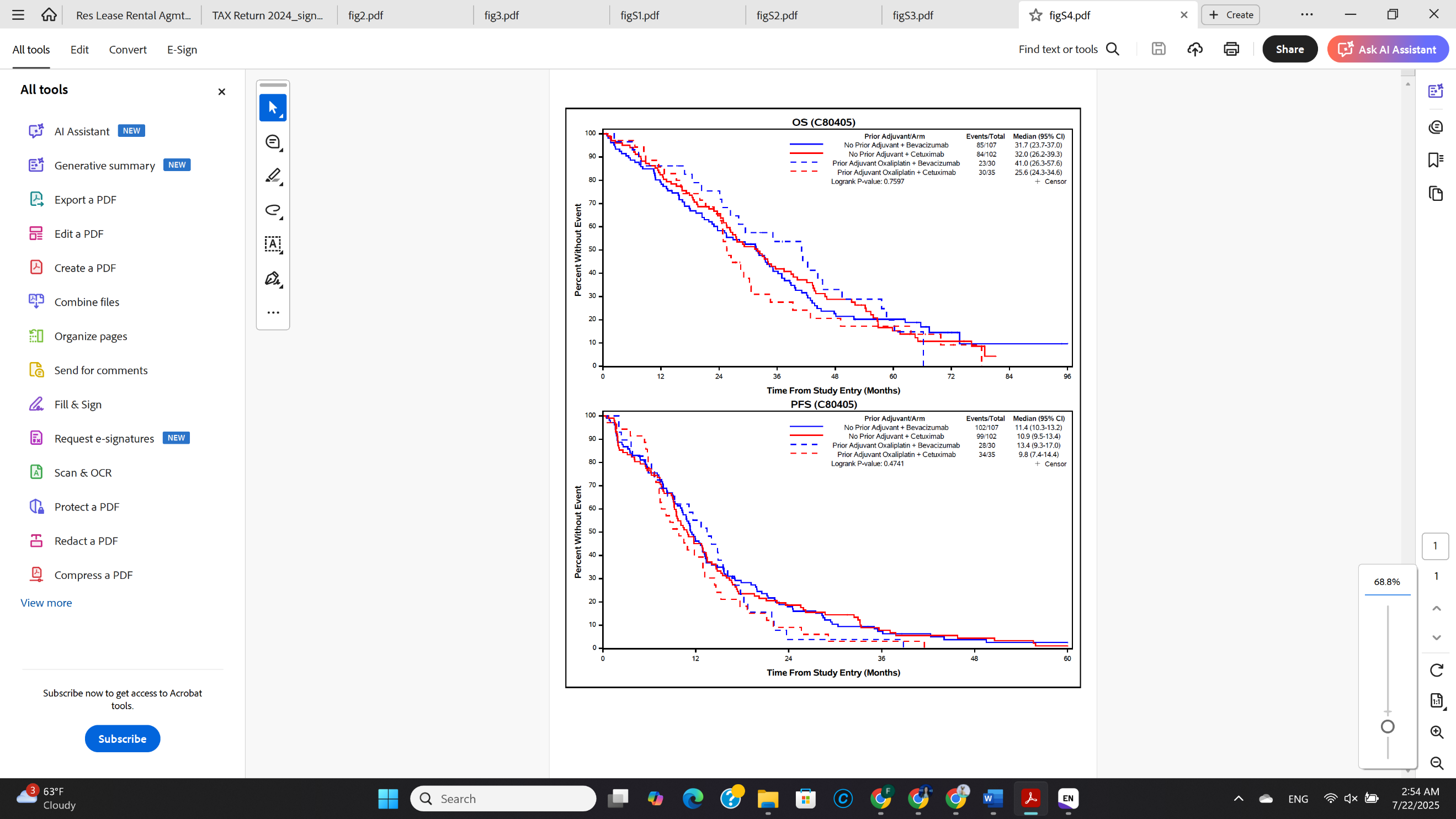

Supplement: Supplemental Material [file NIHMS2158207-supplement-Supplemental_Material.docx]
